# Mining the Gems of a Web-Based Mindfulness Intervention: Qualitative Analysis of Factors Aiding Completion and Implementation

**DOI:** 10.2196/37406

**Published:** 2022-10-05

**Authors:** Muskan Yadav, Sandra Neate, Craig Hassed, Richard Chambers, Sherelle Connaughton, Nupur Nag

**Affiliations:** 1 Department of General Practice Monash University Melbourne Australia; 2 Neuroepidemiology Unit Melbourne School of Population and Global Health The University of Melbourne Melbourne Australia; 3 Monash Centre for Consciousness and Contemplative Studies Monash University Melbourne Australia

**Keywords:** digital intervention, health education, mindfulness, online learning, behavior change, mental health, mental well-being, physical well-being, meditation, health promotion, digital health, eHealth, thematic analysis, attrition, participation, involvement, engagement, attitude, perspective, patient education, e-learning, user feedback

## Abstract

**Background:**

Digital health interventions provide a cost effective and accessible means for positive behavior change. However, high participant attrition is common and facilitators for implementation of behaviors are not well understood.

**Objective:**

The goal of the research was to identify elements of a digital mindfulness course that aided in course completion and implementation of teachings.

**Methods:**

Inductive thematic analysis was used to assess participant comments regarding positive aspects of the online mindfulness course Mindfulness for Well-being and Peak Performance. Participants were aged 18 years and older who had self-selected to register and voluntarily completed at least 90% the course. The course comprised educator-guided lessons and discussion forums for participant reflection and feedback. Participant comments from the final discussion forum were analyzed to identify common themes pertaining to elements of the course that aided in course completion and implementation of teachings.

**Results:**

Of 3355 course completers, 283 participants provided comments related to the research question. Key themes were (1) benefits from the virtual community, (2) appeal of content, (3) enablers to participation and implementation, and (4) benefits noted in oneself. Of subthemes identified, some, such as community support, variety of easily implementable content, and free content access, align with that reported previously in the literature, while other subthemes, including growing together, repeating the course, evidence-based teaching, and immediate benefits on physical and mental well-being, were novel findings.

**Conclusions:**

Themes identified as key elements for aiding participant completion of a mindfulness digital health intervention and the implementation of teachings may inform the effective design of future digital health interventions to drive positive health behaviors. Future research should focus on understanding motivations for participation, identification of effective methods for participant retention, and behavior change techniques to motivate long-term adherence to healthy behaviors.

## Introduction

Web-based educational interventions have the potential to be implemented for disease prevention, positive behavior change, and self-managed well-being; they are scalable and can be disseminated widely [1-4]. In addition, the digital format enables participants to schedule and plan their learning, provides relative anonymity for open questioning and avoidance of perceived stigmatization for topics such as mental health, and minimizes physical accessibility and financial barriers [5,6]. During the COVID-19 pandemic, restrictions on in-person interactions were introduced to minimize virus spread. Physical barriers to education and demand for self-help courses led to a rapid change and increase in digital communication and learning [7]. Moreover, a global rise in mental health conditions resulting from the pandemic [8,9] prompts an immediate need for efficacious interventions that may increase resilience and well-being.

Mindfulness is a form of mental training that has been effectively used to improve health outcomes in clinical and nonclinical populations [10-12]. Defined as the nonjudgmental awareness of the environment, one’s body, and one’s own thoughts and feelings in the present moment, it can be developed through a range of formal (mindfulness meditation), informal (being mindful in daily life), and cognitive (reflecting on various aspects of mindfulness—for example, acceptance and letting go) practices. Meta-analyses indicate that mindfulness-based interventions (MBIs) facilitate healthy lifestyle change [3]; reduce stress, depression, and anxiety; and improve resilience, cognitive function, sleep, and general well-being [13].

Digital MBIs aim to enable health-based behavior change through real-time or asynchronous participation in self-guided or facilitator-supported content. MBIs have traditionally been enacted in-person where direct, personal, and reflective quality reinforces learning; these factors may be lacking in a digital format, bringing to question the effectiveness of digital MBIs. Studies directly comparing online versus in-person delivery of MBIs have reported similar benefits on measured health outcomes [14], and many studies have reported on the effectiveness of online delivery to reduce perceived stress and improve mindfulness, mental health–related outcomes, and well-being [15]. Key success components of web-based interventions include program content, multimedia resources, interactive online activities, and guidance or supportive feedback [16], while challenges include selecting and understanding the audience, effective learning, and defining learning objectives [1,17]. High attrition rates and low adherence are common. In a systematic review of studies of smartphone apps for depressive symptoms, attrition was near 50% but lower in studies offering human feedback and in-app mood monitoring [18]. Low adherence is associated with reduced intervention efficacy and possibly related to a combination of characteristics of the condition addressed by the intervention, the user, and the intervention itself [4,19]. Understanding components that aid in course completion and implementation of learned practices may inform methods to minimize some of the challenges in web-based educational interventions for positive behavior change.

Mindfulness for Well-being and Peak Performance (MWPP) is a massive online open course (MOOC) which, based on user reviews on Class Central, has been ranked in the top MOOCs globally since the year it began. A study of 2105 participants who completed MWPP found significant improvement in mindfulness, perceived stress, and work engagement [20]. Given the popularity and efficacy of MWPP, we qualitatively analyzed participants’ free text comments on the course’s final learner forum asking “What were the gems of the course?” to explore the positive aspects of the course. Having explored all forum comments, two research questions arose: “What course components aided course completion and implementation of learned practices?” and “How did participants learn mindfulness, and what outcomes resulted from this?” In this study, the research question regarding factors assisting course completion and implementation of learnings was explored.

## Methods

### Digital Mindfulness Intervention

The MWPP course was developed in 2015 and delivered digitally thrice annually until the end of 2021 by CH and RC. The course is hosted on the FutureLearn platform and open for free registration globally. Registered participants were sent weekly email reminders during the 4-week period prompting them to complete course modules.

The course recommended a commitment of 3 hours per week to cover topics that build skills and knowledge progressively. Content was delivered live in English via short videos, curated articles, downloadable guided meditations, quizzes on core content, links to further resources, moderated discussion forums, and weekly feedback videos. Discussion forums, based on weekly topics and designed to encourage self-reflective learning and discussion between participants, were facilitated by SC and another mentor with the support of educators CH and RC. Content for weekly feedback videos was based on prevalent participant questions and insights from discussion forums. At the end of the 4-week course, participants had an additional 2 weeks to complete the modules, without live mentoring. The course was free for this 6-week period with an optional one-time fee charged for unlimited access to content thereafter.

### Participants and Data Collection

Participants self-enrolled in the 16th run of the MWPP course, delivered from March to April 2020. Of 23,932 enrolled participants, 18,080 participants began the course and 3335 (18.4%) completed at least 90% of the modules. Of MWPP course completers, 527 responded to the final discussion forum questions “Have you joined this course before, and if so, what brings you back?” and “What are the gems for you from this course?” (data from responses to the latter were deidentified and extracted for analysis).

### Ethics Approval

This study was approved by the Monash University Human Research Ethics Committee (ID 18105).

### Analysis

Participants’ comments were analyzed using inductive thematic analysis, which identifies, analyses, and reports patterns within qualitative data in a structured format [21]. This form of analysis was selected given the content of the data varied and thematic analysis can detect patterns that facilitate understanding. Responses to the question of interest were securely exported into an Excel (Microsoft Corp) document.

The analysis team comprised MY, NN, and SN. All 527 participant responses to the final discussion forum question were independently reviewed in an iterative process to aid familiarization with the data and develop initial codes (NN and SN). Comments in languages other than English and from educators and mentors were excluded. From initial codes, NN and SN identified 2 primary and independent categories from the data: course-related (digital health interventions) codes versus value and impact-related codes. These categories were derived without preconceived ideas of categories or theoretical perspectives. A total of 283 participant comments related to the first overarching category of digital health interventions. These 283 comments were reanalyzed and recoded, and themes that contained a central organizing concept were identified and named (NN and MY). Analysis of comments relating to the second category are reported elsewhere [22].

The data were reviewed to ensure themes were appropriately named and reflected the data. Detailed records of researcher meetings (NN and MY) were maintained by MY to enhance transparency. Themes are presented in the context of a digital health program evaluation framework [23] and subthemes illustrated with verbatim quotes to enable readers to assess the validity and fittingness of researcher interpretations [24].

## Results

### Participant Characteristics

Of the course completers, 15.8% (527/3335) responded to the final discussion forum question. Of the comments, 53.7% (283/527) were relevant to the research question. Due to FutureLearn’s privacy policy, it was not possible to match comments to individual participant demographics.

### Qualitative Findings

Four themes and 21 subthemes were identified for components of the course that aided in course completion and implementation of teachings:

1. Benefits from the virtual community
  - Mentor/educator feedback
  - Shared experiences
  - Support
  - Growing together
2. Appeal of content
  - Variety of format
  - Optional content
  - Unrestricted access
  - Visual aids
  - Life examples
  - Ease of implementation
  - Delivery by educators
3. Enablers to participation and implementation
  - Own pace
  - Free access
  - Option to repeat course
  - Evidence-based teaching
  - New perspectives
4. Benefits noted in oneself
  - Knowledge gained
  - Stress management
  - Behavior change
  - Mind and body benefits
  - Increased ability to cope

Selected participant quotes embodying subthemes are presented in text, and a representative quote for each theme tabulated (Table 1).

**Table 1:** Themes and illustrative quotes.

Theme	Illustrative quote
Benefits from the virtual community	“Having the opportunity to exchange ideas with other learners helps a lot in the process, as you learn from them also and share ideas and feelings.”
Appeal of content	“I am very impressed with the course and extra PDF files and videos. From the first week, I was making changes in my life.”
Enablers to participation and implementation	“Mindfulness is so helpful right now. I had a sense of how that would be but it’s only when you actually study and practice mindfulness at a time when all around you is noise, panic, and confusion that you truly realize its importance.”
Benefits noted in oneself	“Once again, many thanks for this course as it’s helped me to ease my worries working with COVID-19 patients. I feel more relaxed and less anxious. Worry is a just a thought. With meditation, I can change my thoughts for a positive one. And being connected with the present helps me to perform better in my job.”

### Benefits from the Virtual Community

Participants appreciated and realized positive outcomes from feeling part of and interacting with members of the course’s virtual community. This included feeling guided and engaged due to mentor feedback, feeling supported, experiencing comradery with fellow participants, and growing by virtue of reflective interactions with other learners. This theme highlights principles of acceptability and adoption as well as technology and function within the evaluation framework [23], with 4 subthemes identified.

#### Mentor and Educator Feedback

Mentoring was valuable as it helped stay on the right path, with individual concerns addressed and attended to. Educator feedback also enhanced the ability of the course to be engaging as the weekly feedback videos imparted a sense of real-time interaction.

As ever, the “live” aspect of this course keeps me engaged.... I look forward to Craig and Richard’s feedback videos.

#### Shared Experiences

Through the online interactions with fellow learners who shared similar views, participants felt connected. The course also provided a platform for participant challenges to be validated, and they could find solace in that fellow learners were experiencing the same.

It’s been especially meaningful to connect with like-minded souls across the world, with all of us sharing awareness and compassion.

#### Support

The support provided by educators, mentors, and fellow learners seemed to facilitate the practice of mindfulness taught in the course and aid in other challenging life situations.

Thank you to everyone here: educators, mentors, and learners. Your knowledge and comments help go on with my practice, go through difficult times, and situations in my life.

#### Growing Together

Participants reported that through gaining insight from reading others’ views on the discussion forum and by sharing and reflecting on their own experiences, their learning was enhanced.

Being able to read and comment on the “forum” is a great plus. Having the opportunity to exchange ideas with other learners helps a lot in the process, as you learn from them also and share ideas and feelings.

### Appeal of Content

Theme 2, appeal of content, additionally reflected principles of acceptability, adoption, technology, and function [23]. Within this theme, 7 subthemes were identified in which participants noted course content features that were appealing and facilitated engagement in practice and learning. These included multimodal content, optional additional learning materials, relatable life examples, and clear and humorous course delivery.

#### Variety of Format

The multimodal nature of the resources helped sustain participant interest in the material, continue to complete the course, and implement mindfulness in practice.

I am very impressed with the course and extra PDF files and videos. From the first week I was making changes in my life.

#### Optional Content

Emphasis on autonomy and ability to choose the amount, type, and extra content they engaged with was valued.

I would say the entire course is a gem! The way it gave short, open insights with the possibility of expansion and a view to more learning (videos, articles, abstracts, meditations).

#### Unrestricted Access

Unrestricted access to course content throughout the duration of the course was considered appealing as it enhanced equitable access to all resources and satisfaction with the course and enabled participants to revisit lessons and thereby consolidate knowledge at a user-defined pace.

The resources in this course and the way it is delivered has really been useful. I have been able to go back and revisit sections to consolidate my understanding.

#### Visual Aids

The provision of visual aids in the course improved engagement, accessibility, and understanding, especially for people who did not speak English as their first language.

I am Spanish-speaking and being able to study in English, at my own pace, in a relaxed tone and with visual help in the form of videos is a great experience for me.

#### Life Examples

The types of examples and approaches provided by the educators were relatable to participants and led to feelings that the content was personalized and applicable to their own life.

It felt like, at times, that this course was designed specifically for me. It felt like I was having a conversation about my life.

#### Ease of Implementation

Mindfulness practices were appealing as they were easily implementable. Practices varied in duration, and educators provided examples of mindfulness that were practical and applicable in daily life and activities. The variable duration of practices enhanced accessibility as participants could tailor practices to suit their circumstances.

The practices are as short or as long as you want them to be giving you just enough time to change your mindset at that moment to help cope with stress or just the rest of your day.

#### Delivery by Educators

Participants appreciated the delivery of content through videos and feedback-aided learning, effective educator communication, and content delivered with humor.

The educators are really good at communicating the material in an easy-to-understand way and make it interesting and easy to practice.

### Enablers to Participation and Implementation

Theme 3 encompassed aspects of the course that facilitated continued participation in the course and implementation of mindfulness practice, aligning with principles of accessibility, acceptability, and adoption [23]. Five subthemes were identified: ability to work through content at one’s own pace, free access to course content, option to repeat course for consolidation of learning, evidence-based teaching, and new perspectives of mindfulness gained.

#### Own Pace

Self-paced learning was a key enabler for participation and thus acceptability of the course. The flexible form of learning enabled accessibility, removing potential barriers of different learning speeds and capabilities, time availability, and motivation.

It was at times hard for me to keep up here. I started late, and then COVID-19 hit us all with a blast. But I’ve made it!

#### Free Access

Free access to the course was important for accessibility, particularly relevant and valued during times of hardship. During the COVID-19 pandemic, emotional and financial distress were widespread across societies; having free access to resources was an enabler for participation in the course and implementation of mindfulness practice.

Thank you for making the resources available for us for free. A bit more than 4 weeks into quarantine, jobless, and lost a few family members, and I know this course helped me going through all of that.

#### Option to Repeat Course

Participants reported that reenrolling to repeat the course was useful for implementation of mindfulness practice as it allowed reestablishment and building of knowledge and skills learned previously and revived motivation for practice.

I have done this course before. I find it really useful to refresh and motivate myself. As I cannot take it all in during the 4 weeks, a revisit allows me to remind myself of what I have learned previously and to build on my understanding.

#### Evidence-Based Teaching

Delivery of evidence-based content and knowledge allowed for increased acceptability of the practices, enhanced motivation to adopt mindfulness, and increased participant ease.

The gem for me was the evidence re mindfulness practice and improved cognitive ability, improved health outcomes, and improved quality of life. This wealth of evidence is helpful to maintain motivation for mindfulness practice.

#### New Perspectives

Through learning and practicing the course content, participant perspectives on the value of mindfulness practice for embracing and managing life challenges were either reinforced or new perspectives revealed.

Mindfulness is so helpful right now. I had a sense of how that would be but it’s only when you actually study and practice mindfulness at a time when all around you is noise, panic, and confusion that you truly realize its importance.

### Benefits Noted in Oneself

Theme 4 aligned with the evaluation framework principles of safety and quality, health outcomes, and acceptability and adoption [23]. The 5 subthemes revealed positive outcomes for improved knowledge, stress management, positive behavior change, and improved well-being.

#### Knowledge Gained

Participants became aware of certain habitual behaviors. Through course explanations provided regarding these behaviors and why they may be ineffective, participants gained skills that they could use to recognize and change their behaviors.

Uni task. This section was the one that resonated most with me. I was happy in the belief that I could multitask, so it was when Craig and Richard were explaining what actually happens that I recognized some of the issues and barriers I face on a regular basis. I am going to make an effort to focus on just one task at a time.

#### Stress Management

The course taught participants practical skills of how to be mindful in different situations and environments that could be applied long term. Their immediate implementation of mindfulness resulted in identifiable positive outcomes including managing stress, changing thoughts from negative to positive, and improved performance.

Once again, many thanks for this course as it’s helped me to ease my worries working with COVID-19 patients. I feel more relaxed and less anxious. Worry is a just a thought. With meditation I can change my thoughts for a positive one. And being connected with the present helps me to perform better in my job.

#### Behavioral Change

Participants acknowledged that being mindful in performing daily tasks and actions helped them to focus, understand and enjoy life, and achieve more. They noted changes in behavior with an ability to regulate demands on self and others, let go of negative emotions, and accept limitations to maintain well-being.

Perception, letting go, acceptance, and presence of mind are now my mantra. I’ve learned to be less demanding of myself and others, this does not mean that I have low expectations, it simply signifies that I’m more realistic about what to expect. I’ve also learned to let go of those harmful feelings of rage, anger, and disappointment. Being mindful, for me, also taught me that that’s ok to say no, to respect and accept my limitations, and to safeguard my well-being.

#### Mind and Body Benefits

Daily mindfulness resulted in benefits to participants’ mental and physical well-being, with many reporting feelings of positivity, as well as improved concentration, sleep, and fitness, reflecting improved health outcomes.

This course has made me feel more positive, I am loving the meditations, and it has helped me improve my fitness and focus on the here and now.

#### Increased Ability to Cope

Improved coping skills were described as a positive outcome, gained through learning concepts of perception, letting go, acceptance, and presence of mind, which could be understood and predicted to be applicable to manage strong emotions arising in the future.

This course was timely for me as it helped me push through the challenge, progress, and pick me up during the pandemic shutdown and work from home experience.

## Discussion

### Principal Findings

High participant attrition and low adherence to practice are common in digital health interventions [4,18,19]. Our qualitative study of participant feedback from a web-based mindfulness course identified 4 key elements that aided course completion and implementation of teachings: (1) benefits from the virtual community, (2) appeal of content, (3) enablers to participation and (4) benefits noted in oneself. Together the themes embodied safety and quality, accessibility, acceptability and adoption, health outcomes, and technology and function aspects of an evaluation framework [23], highlighting the effectiveness of the MWPP as a digital health intervention.

### Comparison to Prior Work

Various challenges have been identified in creating effective digital behavior change interventions (DBCIs), particularly in relation to understanding and promoting engagement [17]. These challenges include developing DBCIs that are person-centered and iterative and meet user requirements and establishing what constitutes effective engagement for a DBCI [25]. Through course elements like mentoring and feedback videos, a very person-centered, iterative, and supportive approach for the individual and the learning community were important elements in the success and popularity of the MWPP course. Further, engagement with an online learning platform does not necessarily translate into engagement with behavior change, nor does it mean that short-term behavior changes as a part of the program will translate into longer term healthy behaviors [26]. Mixed method approaches including qualitative data provide important insights into why participants find a DBCI effective or ineffective [27].

Benefits gained from a virtual community were reported to be achieved through interaction with mentors and fellow learners in the discussion forums and through educator feedback. Our findings align with prior studies showing support provided by therapists and other participants are significant factors in the experience of participating in MBIs [4]; support through existence of another person or social presence can influence accountability and thus adherence to the course [28]. Other qualitative studies on mindfulness reflect the notion of group support to be valuable [14], with theoretical research suggesting it is the strongest predictor of behavior change [28]. It is interesting to note that comments in this theme reflect perceived support, with research showing this perception enhances coping and self-esteem [28]. A sense of connectedness and validation of feelings by sharing experiences and gaining and reflecting on learning and practice together were novel concepts for online interventions. These suggest the value of group interaction and discussion for enhancing motivation for continued learning, consolidation of teachings, and confidence in implementation of practice.

The multimodal and optional course content were valued and conducive to course completion, in accord with previous studies showing that appeal of intervention features improves the quality of the user experience and has strong influence on initiating and sustaining user engagement [29]. Unlimited and free access to content, flexibility of participation in the learning materials, and access to the benefits of discussion forums and educator feedback were also identified as aiding in course completion. Previous research has shown time constraints to be one of the key barriers to engaging in MBIs, and reducing this through features such as optional additional resources grants autonomy over amount of time invested and is favorable to participants [30]. Novel aspects identified in course content were inclusion of visual aids, which enabled accessibility to nonnative English speakers, and easy-to-understand delivery of concepts by educators. This provides valuable insight for a means to mitigate other potential language, reading, and listening barriers. Content that aided in implementation of mindfulness practice were provision of multiple options and providing life examples with which learners could identify.

Enablers to participation and implementation included provision of evidence-based content, which has been shown in prior research to improve outcomes by enabling acceptance in academically minded groups [31]. Self-pacing was a valued aspect of this course, and this is supported by the theory of self-determination—namely, that self-efficacy and autonomy increase successful implementation [32]. Novel findings within this theme were participants reporting repetition of the course provided motivation and enabled reestablishment and maintenance of skills and participants preconceptions of mindfulness changing because of course content. These aspects may be important for sustained long-term adherence to behavior change.

Benefits noted in oneself identified an increase in self-regulation (consciously managing emotions and behaviors), which is a recognized change induced by mindfulness meditation [33]. There is strong evidence shown in the literature that self-regulation facilitates behavior change as it improves participants’ innate motivation and focus, which can assist in retaining and efficiently learning material leading to lasting changes [33].

### Strengths and Limitations

Limitations of the study are acknowledged. First, of participants who commenced the MWPP course, only 18% completed at least 90%. Reasons for and timing of dropout from the course were not collected, although for MOOCs, an 18% completion rate is quite high. These details, as well as information on motivations for commencing the course, may provide further insight to key components for participant retention. Second, of the MWPP course completers, 16% contributed to the data. Our results may not be representative of all course completers; however, data from a large sample are not crucial for qualitative analyses [34]. Methods to increase participant feedback may include mandatory survey completion at intervals to progress to subsequent sections and financial incentives for survey completion. A third limitation noted is the free-text nature of the feedback question, which directed participants to specifically comment on positive aspects of the course. Reframing the feedback question to query positive and negative aspects as well as course components that facilitated and deterred completion and implementation of teachings may reveal greater depth of knowledge.

The strengths of our study include the data being drawn from a diverse and significant sample of engaged participants, many of whom were experienced with the course. Thematic analysis allowed for a range of relevant issues to be highlighted.

### Future Directions

Future research should focus on understanding motivations for participation in mindfulness and identifying effective methods for participant retention and behavior change techniques to motivate long-term adherence to healthy behaviors.

This may include research on behavior change techniques to motivate long-term adherence of healthy behaviors. Together, these questions may address challenges in the design of effective digital interventions for self-managed improved well-being.

For future research, the established mindfulness techniques used in this course can be explored to allow for formal reporting and use in other behavior change interventions.

### Conclusion

Supportive community, appealing content, enablers to participation and implementation, and positive self-benefits were identified as core aspects aiding in completion of an online mindfulness course and implementation of teachings. These insights may aid future digital health intervention designers to optimize elements that lead to optimal learning and effective implementation of health behaviors.

## Abbreviations

DBCI: digital behavior change intervention
MBI: mindfulness-based intervention
MOOC: massive open online course
MWPP: Mindfulness for Well-being and Peak Performance

## References

[1] Mrazek AJ, Mrazek MD, Cherolini CM, Cloughesy JN, Cynman DJ, Gougis LJ, Landry AP, Reese JV, Schooler JW (2019). The future of mindfulness training is digital, and the future is now. Curr Opin Psychol.

[2] Taylor H, Strauss C, Cavanagh K (2021). Can a little bit of mindfulness do you good? A systematic review and meta-analyses of unguided mindfulness-based self-help interventions. Clin Psychol Rev.

[3] Sancho M, De Gracia M, Rodríguez RC, Mallorquí-Bagué N, Sánchez-González J, Trujols J, Sánchez I, Jiménez-Murcia S, Menchón JM (2018). Mindfulness-based interventions for the treatment of substance and behavioral addictions: a systematic review. Front Psychiatry.

[4] Murray E (2012). Web-based interventions for behavior change and self-management: potential, pitfalls, and progress. Med 2 0.

[5] Christensen H, Bennett-Levy J, Richards D, Farrand P, Christensen H, Griffiths K, Kavanagh D (2010). Increasing access and effectiveness: using the internet to deliver low intensity CBT. Oxford Guide to Low Intensity CBT Intervention.

[6] Rochlen AB, Zack JS, Speyer C (2004). Online therapy: review of relevant definitions, debates, and current empirical support. J Clin Psychol.

[7] Mheidly N, Fares MY, Fares J (2020). Coping with stress and burnout associated with telecommunication and online learning. Front Public Health.

[8] Santomauro D, Mantilla Herrera A, Shadid J, Zheng P, Ashbaugh C, Pigott D (2021). Global prevalence and burden of depressive and anxiety disorders in 204 countries and territories in 2020 due to the COVID-19 pandemic. Lancet.

[9] Torales J, O'Higgins M, Castaldelli-Maia JM, Ventriglio A (2020). The outbreak of COVID-19 coronavirus and its impact on global mental health. Int J Soc Psychiatry.

[10] Liu Y, Li I, Hsiao F (2021). Effectiveness of mindfulness-based intervention on psychotic symptoms for patients with schizophrenia: a meta-analysis of randomized controlled trials. J Adv Nurs.

[11] Scott-Sheldon LAJ, Gathright EC, Donahue ML, Balletto B, Feulner MM, DeCosta J, Cruess DG, Wing RR, Carey MP, Salmoirago-Blotcher E (2020). Mindfulness-based interventions for adults with cardiovascular disease: a systematic review and meta-analysis. Ann Behav Med.

[12] Galante J, Friedrich C, Dawson AF, Modrego-Alarcón M, Gebbing P, Delgado-Suárez I, Gupta R, Dean L, Dalgleish T, White IR, Jones PB (2021). Mindfulness-based programmes for mental health promotion in adults in nonclinical settings: a systematic review and meta-analysis of randomised controlled trials. PLoS Med.

[13] Goldberg SB, Riordan KM, Sun S, Davidson RJ (2021). The empirical status of mindfulness-based interventions: a systematic review of 44 meta-analyses of randomized controlled trials. Perspect Psychol Sci.

[14] Klatt M, Bawa R, Gabram O, Westrick A, Blake A (2021). Synchronous mindfulness in motion online: strong results, strong attendance at a critical time for health care professionals (HCPs) in the COVID era. Front Psychol.

[15] Zhang Y, Xue J, Huang Y (2020). A meta-analysis: internet mindfulness-based interventions for stress management in the general population. Medicine (Baltimore).

[16] Barak A, Klein B, Proudfoot JG (2009). Defining internet-supported therapeutic interventions. Ann Behav Med.

[17] Michie S, Yardley L, West R, Patrick K, Greaves F (2017). Developing and evaluating digital interventions to promote behavior change in health and health care: recommendations resulting from an international workshop. J Med Internet Res.

[18] Torous J, Lipschitz J, Ng M, Firth J (2020). Dropout rates in clinical trials of smartphone apps for depressive symptoms: a systematic review and meta-analysis. J Affect Disord.

[19] Ryan C, Bergin M, Wells JS (2017). Theoretical perspectives of adherence to web-based interventions: a scoping review. Int J Behav Med.

[20] Bartlett L, Buscot M, Bindoff A, Chambers R, Hassed C (2021). Mindfulness is associated with lower stress and higher work engagement in a large sample of MOOC participants. Front Psychol.

[21] Braun V, Clarke V (2006). Using thematic analysis in psychology. Qual Res Psychol.

[22] Neate SL, Reece JC, Hassed C, Chambers R, Connaughton S, Nag N (2022). A qualitative analysis of free text comments of participants from a massive open online mindfulness course. Front Public Health.

[23] (2016). Evaluation resource guide: allied health telehealth capacity building project. Queensland Health.

[24] Beck CT (1993). Qualitative research: the evaluation of its credibility, fittingness, and auditability. West J Nurs Res.

[25] Yardley L, Morrison L, Bradbury K, Muller I (2015). The person-based approach to intervention development: application to digital health-related behavior change interventions. J Med Internet Res.

[26] Mohr DC, Schueller SM, Montague E, Burns MN, Rashidi P (2014). The behavioral intervention technology model: an integrated conceptual and technological framework for eHealth and mHealth interventions. J Med Internet Res.

[27] Morrison LG, Hargood C, Lin SX, Dennison L, Joseph J, Hughes S, Michaelides DT, Johnston D, Johnston M, Michie S, Little P, Smith PW, Weal MJ, Yardley L (2014). Understanding usage of a hybrid website and smartphone app for weight management: a mixed-methods study. J Med Internet Res.

[28] Santarossa S, Kane D, Senn CY, Woodruff SJ (2018). Exploring the role of in-person components for online health behavior change interventions: can a digital person-to-person component suffice?. J Med Internet Res.

[29] Short C, Rebar A, Plotnikoff R, Vandelanotte C (2015). Designing engaging online behaviour change interventions: a proposed model of user engagement. Euro Health Psychol.

[30] Banerjee M, Cavanagh K, Strauss C (2017). A qualitative study with healthcare staff exploring the facilitators and barriers to engaging in a self-help mindfulness-based intervention. Mindfulness (NY).

[31] Moore S, Barbour R, Ngo H, Sinclair C, Chambers R, Auret K, Hassed C, Playford D (2020). Determining the feasibility and effectiveness of brief online mindfulness training for rural medical students: a pilot study. BMC Med Educ.

[32] Ng JYY, Ntoumanis N, Thøgersen-Ntoumani C, Deci EL, Ryan RM, Duda JL, Williams GC (2012). Self-determination theory applied to health contexts: a meta-analysis. Perspect Psychol Sci.

[33] Schuman-Olivier Z, Trombka M, Lovas DA, Brewer JA, Vago DR, Gawande R, Dunne JP, Lazar SW, Loucks EB, Fulwiler C (2020). Mindfulness and behavior change. Harv Rev Psychiatry.

[34] Marks D, Yardley L (2004). Research Methods for Clinical and Health Psychology, First Edition.

